# Analysis of multiple organ function damage in patients with severe COVID-19 pneumonia

**DOI:** 10.5937/jomb0-41502

**Published:** 2023-08-25

**Authors:** Shiyan Feng, Fengxin Wang, Weibo Wu, Yinfeng Li, Chuming Chen, Jianming Li, Mengli Cao, Ling Peng, Peiyan Zhang, Fuxiang Wang

**Affiliations:** 1 Second Hospital Affiliated to Southern University of Science and Technology, Shenzhen Third People's Hospital, Shenzhen, Guangdong Province, China

**Keywords:** severe cases, coronavirus disease 2019 (COVID-19) pneumonia, organ function index, teški slučajevi, coronavirus oboljenje 2019 (COVID-19) pneumonija, indeks funkcije organa

## Abstract

**Background:**

This study aims to analyze the changes and significance of organ function indices in patients with severe Coronavirus Disease 2019 (COVID-19) pneumonia for prediction of major organ damages and guiding treatment schemes.

**Methods:**

63 patients with severe COVID-19 pneumonia were selected as the severe group and 73 patients with mild syndromes were selected as the mild group. SAS9.4 software was used for statistical analysis of the data.

**Results:**

Levels of ALT, AST, cTnI, Cr, PT, APTT and Ddimer of the severe group were significantly higher while PLT was lower than those of the mild group. The data of all quantitative variables were converted into categorical variables. Significantly higher levels of AST, ALB, D-dimer and higher proportion of bilateral lung involvement were observed from the severe group comparing to those in the mild group, while the difference in the other indices between the two groups was insignificant in statistical perspective.

**Conclusions:**

There are significant differences in the levels of multiple organ function indices between the severe group and the mild group of patients with COVID-19 pneumonia infection. Through examining the relevant indices, conditions of patients' multiple organ function damage could be predicted and used as guidance of treatment.

## Abstract (sr)

**Uvod:**

Ova studija ima za cilj da analizira promene i značaj indeksa funkcije organa kod pacijenata sa teškom pneumonijom od korona virusne bolesti 2019 (COVID-19) radi predviđanja velikih oštećenja organa i vođenja šema lečenja.

**Metode:**

63 pacijenta sa teškom COVID-19 pneumonijom odabrana su kao teška grupa, a 73 pacijenta sa blagim sindromom izabrana su kao laka grupa. Za statističku analizu podataka korišćen je softver SAS9.4.

**Rezultati:**

Nivoi ALT, AST, cTnI, Cr, PT, APTT i D-dimera u teškoj grupi su bili značajno viši dok je PLT bio niži od onih u blažoj grupi. Podaci svih kvantitativnih varijabli pretvoreni su u kategorijske varijable. Značajno viši nivoi AST, ALB, Ddimera i veći udeo bilateralnog zahvatanja pluća primećeni su u teškoj grupi u poređenju sa onima u blažoj grupi, dok je razlika u ostalim indeksima između dve grupe bila beznačajna u statističkoj perspektivi.

**Zaključak:**

Postoje značajne razlike u nivoima indeksa funkcije više organa između teške grupe i blage grupe pacijenata sa infekcijom pneumonijom COVID-19. Ispitivanjem relevantnih indeksa mogu se predvideti stanja višeorganskih funkcija pacijenata i koristiti kao smernica za lečenje.

## Introduction

Since the start of the outbreak of Coronavirus Disease 2019 (COVID-19) infection from Dec 2019 in Wuhan, China, it now becomes a worldwide pandemic and affects almost everyone in the world. According to the latest statistics of National Health Commission of the People's Republic of China, there were 84522 confirmed cases and 4645 deaths being reported in China on 24 May 2020. In total, there were more than 5.1 million confirmed cases and 333401 deaths reported from 216 countries, areas or territories in the worldwide as released by World Health Organization on 24 May 2020. (www.who.int)

COVID-19 is highly infectious with high speed of transmission and high mortality rate [1]. Its routes of transmission from person to person include aerosol, fecal-oral, ocular surface and secretions and etc [1] [2]. It has been reported that mortality rate is higher in the aged patients and those with chronic underlying diseases such as diabetes and cardiovascular diseases [3] [4]. Typical symptoms of COVID-19 infection include fever, coughing, muscle ache, fatigue, chest pain and etc. [5]. Various studies investigated the changes in the level of blood routine indices and sequential organ failure assessment scores to assess the infection progression in the patients [6] [7] [8]. Moreover, SARS-CoV-2 viral infection would damage various major organs and tissues including the liver, the kidney, the heart and the blood, and is able to develop to multiple fetal organ failures leading to the death of the patients [5] [6] [9] [10]. Deaths of COVID-19 infected kidney transplant recipients were also reported, showing the influence of this virus on major organ functions [11]. However, current treatment measures taken towards COVID-19 infection are still mostly supportive and unspecific. Alternative treatment measures have been tested. Improvements of the clinical conditions could be seen from critically ill patients after receiving antibody-containing covalescent plasma transfusion [7]. However, further thorough study is still needed on the effectiveness of this treatment measure. Therefore, it is spectacularly important to monitor the relevant indices of organ functions to predict the severity of the infection for patient protection and corresponding treatments. In this study, we analyzed multiple organ function indices of 136 COVID-19 pneumonia patients and compared the differences in the index levels between the severely ill patients and the patients with mild syndromes to understand the progress and the outcome of the COVID-19 infection for better disease control and more effective treatment.

## Materials and Methods

### Patients

From January 2020 to March 2020, among the COVID-19 pneumonia patients charged into The Third People's Hospital of Shenzhen (the designated medical institution for COVID-19 pneumonia treatment in Shenzhen), 63 severe COVID-19 pneumonia patients were selected as the severe group among which 39 patients are male and 24 patients are female, aged from 21 to 86 years old with average age of 61.3 years. 73 mild COVID-19 pneumonia patients were selected as the mild group among which 30 patients are male and 43 patients are female, aged from 1 year 8 months to 79 years old with average age of 40.5 years. The level of severity is classified according to the classification guidance provided by the fifth edition of Novel Coronavirus Pneumonia Diagnosis and Treatment Plan.

### Sample collection and tests

This study is approved by the Ethics Committees from Shenzhen Third People's Hospital (2020-142). The index values of peripheral venous blood samples collected on admission from the two groups of patients were used for retrospective analysis. The biochemical indices and blood routine indices of the blood samples were examined using chemiluminescent method. Patients' lung involvement conditions and the results of color echocardiography were also collected.

### Statistical analysis

SAS9.4 software was used for the statistical analysis of the indexes. Kolmogorov-Smirnov test was adopted to check the normality of the quantitative data. The data following normal distribution is expressed as mean ± standard deviation (x̅±s) while the data not following normal distribution is expressed as median (interquartile range). Inter-group comparison of the normally distributed data was done using Student's t test while the inter-group comparison of the non-normally distributed data was done using Wilcoxon rank-sum test. Inter-group comparison of the qualitative data was done using Chi-square test. Fisher's exact test was applied for those unfitted for Chi-square test. Significant statistical difference is indicated by P<0.05.

## Results

### Patients' epidemiological history and chronic diseases

Based on the information collected, patients in the severe group have higher average age of 61.3 years and more chronic diseases. In the severe group, 44 patients had residential/contact history to Wuhan/Hubei epidemic areas while 4 patients had contact history with confirmed cases. 2 patients had neither been in Wuhan/Hubei or in contact with any confirmed case. 34 patients have at least one type of chronic diseases, including hypertension, diabetes, coronary heart disease, arrhythmia, COPD and hepatitis B. 13 of them have two or more underlying diseases. Six patients turned to critical state and three of them died. Two died cases were with either hypertension or COPD while the third case had no chronic disease history. The other three critical cases had chronic disease history of hypertension or diabetes, or both. Other patients were discharged upon recovery. The average course of disease was 4.9 days while average length of hospitalization was 29 days.

In the mild group, 53 patients had residential/contact history to Wuhan/Hubei epidemic areas. 13 patients had contact history with confirmed cases. 7 patients had no epidemiological history. 13 of the patients have at least one type of chronic diseases, including hypertension, diabetes, emphysema, tuberculosis, hyperlipemia, hepatitis B and cirrhosis at decompensation stage. 2 patients have two underlying chronic diseases. All of the patients in the mild group were discharged upon recovery. The average course of disease was 3.6 days while average length of hospitalization was 19 days.

### Inter-group comparison of the levels of multiple organ function indices

From the results of biochemical test and blood routine tests, significant differences in the levels of multiple organ function indices were seen between the severe group and the mild group. Statistically, positive t or z value represents a higher mean/median value of the index level in the severe group comparing to the mild group, and vice versa. The higher the absolute t or z value, the more different in the index level between the two groups and the lower the P-value (Table 1).

**Table 1:** Comparison of the levels of indices between the severe group and the mild group. Numbers in the bracket represents the range of the particular index detected in the group

Organ function index	Mean/Median<br>Mild Group	Mean/Median<br>Severe Group	t or Z values	P-value
Liver function indices				
ALT (U/L)	20 (14.15–27)	26 (18–35)	2.323	0.02
AST (U/L)	27.75 (22–35)	33 (24.2–45)	2.38	0.017
TBIL (μmol/L)	9.2 (7.7–13.6)	10.5 (7.8–13.6)	0.333	0.739
Alb (g/L)	43.2 (41.65–45.7)	39.7 (37.9–43)	-5.424	<0.001
Kidney function index				
Cr (μmol/L)	59.35 (50–75.2)	68 (55.5–95)	2.695	0.007
Heart function indices				
LDH (U/L)	322 (186–463)	312 (214.5–582)	1.336	0.182
CK-MB (μg/mL)	0.68 (0.22–1.4)	0.78 (0.31–1.42)	0.944	0.345
CK (U/L)	78 (50–119)	90 (52–147)	-0.847	0.397
TnI (ng/mL)	0.01 (0.01–0.01)	0.01 (0.01–0.01)	-2.118	0.034
Blood coagulation indices				
PLT (10^9^/L)	180 (150–217)	164 (127–186)	-3.138	0.002
PT (s)	12.05 (11.4–12.5)	12.2 (11.55–12.75)	1.965	0.049
APTT (s)	34.95 (32.15–38.2)	36.8 (34.35–41.3)	2.312	0.021
D-dimer (μg/mL)	0.34 (0.24–0.47)	0.5 (0.31–0.96)	3.961	<0.001
Immune system function indices				
WBC (10^9^/L)	4.52 (3.43–4.94)	4.74 (3.78–5.8)	1.7	0.089
NEUT (10^9^/L)	2.29 (1.72–3)	2.99 (2.27–4.38)	3.928	<0.001
LYMPH (10^9^/L)	1.39 (0.99–1.74)	0.97 (0.66–1.21)	-4.466	<0.001
PCT (ng/mL)	0.00 (0.03–0.05)	0.06 (0.05–0.08)	-4.522	<0.001
CRP (mg/L)	7.2 (5–17.99)	27 (10.86–52.95)	5.368	<0.001
ESR (mm/L)	20 (12–39)	41.5 (24.5–59.5)	-3.751	<0.001
IL-6 (ng/mL)	12.13 (4.43–17.02)	23.6 (16.63–37.01)	-4.81	<0.001
Lung Function Index				
Oxygenation index	438 (404–466)	345 (270–400)	6.164	<0.001

Significantly lower oxygenation index was detected from the severe group comparing to the mild group. Levels of liver function indices, alanine aminotransferase (ALT) and aspartate aminotransferase (AST), are significantly higher, while level of albumin is lower in the severe group. Elevated concentrations of immune system function indices including C-reactive protein (CRP), procalcitonin (PCT), erythrocyte sedimentation rate (ESR), neutrophil count and interleukin-6 (IL-6) but lower lymphocyte count were also observed in the severe group. Longer plasma prothrombin time (PT) and activated partial prothrombin time (APTT), higher level of D-dimer and lower platelet counts were detected from the severe group. Kidney function index, creatinine (Cr), and heart function index, troponin (TnI), were also increased in patients with severe syndromes. No difference was observed from other indices between the two groups in statistical perspective (Table 1). These results are possibly indicating altered functions of major organs and systems including the heart, the liver, the kidney, the immune system and the blood coagulation system as the severity of the infection increased.

### Inter-group comparison of categorical variables

By converting quantitative variables to categorical variables, the ratio of abnormalities in each category was analyzed between the two groups. Statistically, the proportion of patients with abnormal level of each index were compared between the two groups. Higher x^2^ value represents larger difference between the two groups. Based on the comparison, there were more male patients in the severe group which aligned with the result of previous research study [5]. There are higher proportions of patients with altered lymphocyte count, D-dimer level, albumin level, aspartate aminotransferase level, procalcitonin level, C-reactive protein level, erythrocyte sedimentation rate and interleukin-6 level in the severe group. More patients in the severe group have bilateral lung involvement and altered heart function in the severe group comparing to the mild group. Other indexes are not significantly different in statistical perspective (Table 2). This showed that COVID-19 infection have more extensive influence to organ function indices in the severe group.

**Table 2:** Comparison of the proportion of patients with abnormality in each categorical variable between the severe group and the mild group. Numbers in the bracket: percentages in the group; x^2^: Chi-Square test

Categorical variables	χ^2^	P-value	Mild Group<br> No. of Patients (%)	Severe Group<br> No. of Patients (%)
Gender	5.858	0.016		
Female			43 (58.9)	24 (38.1)
Male			30 (41.1)	39 (61.9)
Bilateral Lung Involvement	7.337	0.007		
No/Left lung/Right Lung			13 (24.07)	3 (5.56)
Yes			41 (75.93)	51 (94.44)
Colored Echocardiography	0.004	0.949		
Normal/Near Normal			7 (100)	17 (89.47)
Involved/Left atrial enlargement			0 (0)	2 (10.53)
Liver function index level				
ALT (U/L)	2.517	0.113		
Within normal range			7 (9.59)	12 (19.05)
Beyond normal range			66 (90.41)	51 (80.95)
AST (U/L)	9.453	0.002		
Within normal range			69 (94.52)	48 (76.19)
Beyond normal range			4 (5.48)	15 (23.81)
TBIL (μmol/L)	0.893	0.345		
Within normal range			65 (89.04)	59 (93.65)
Beyond normal range			8 (10.96)	4 (6.35)
Alb (g/L)	32.245	<0.001		
Within normal range			67 (91.78)	30 (47.62)
Beyond normal range			6 (8.22)	33 (52.38)
Kidney function index level				
Cr (μmol/L)	0.137	0.711		
Within normal range			44 (60.27)	36 (57.14)
Beyond normal range			29 (39.73)	27 (42.86)
Heart function index level				
LDH (U/L)	0.104	0.747		
Within normal range			27 (36.99)	25 (39.68)
Beyond normal range			46 (63.01)	38 (60.32)
CK-MB (ng/mL)	0.568	0.451		
Within normal range			63 (86.3)	57 (90.48)
Beyond normal range			10 (13.7)	6 (9.52)
CK (U/L)	0.592	0.442		
Within normal range			21 (28.77)	22 (34.92)
Beyond normal range			52 (71.23)	41 (65.08)
TnI (ng/mL)	0.006	0.941		
Within normal range			73 (100)	62 (98.41)
Beyond normal range			0 (0)	1 (1.59)
Blood coagulation index level				
PLT (10^9^/L)	3.453	0.063		
Within normal range			67 (91.78)	51 (80.95)
Beyond normal range			6 (8.22)	12 (19.05)
PT(s)	0.035	0.852		
Within normal range			60 (82.19)	51 (80.95)
Beyond normal range			13 (17.81)	12 (19.05)
APTT (s)	2.242	0.134		
Within normal range			61 (83.56)	46 (73.02)
Beyond normal range			12 (16.44)	17 (26.98)
D-dimer (μg/mL)	10.22	0.001		
Within normal range			60 (82.19)	36 (57.14)
Beyond normal range			13 (17.81)	27 (42.86)
Immune system function index level				
WBC (10^9^/L)	0.377	0.539		
Within normal range			51 (69.86)	47 (74.6)
Beyond normal range			22 (30.14)	16 (25.4)
NEUT (10^9^/L)	2.054	0.152		
Within normal range			50 (68.49)	50 (79.37)
Beyond normal range			23 (31.51)	13 (20.63)
LYMPH (10^9^/L)	9.837	0.002		
Within normal range			44 (60.27)	21 (33.33)
Beyond normal range			29 (39.73)	42 (66.67)
PCT(ng/mL)	8.476	0.004		
Within normal range			71 (97.26)	52 (82.54)
Beyond normal range			2 (2.74)	11 (17.46)
CRP (mg/L)	17.831	<0.001		
Within normal range			41 (56.16)	13 (20.63)
Beyond normal range			32 (43.84)	50 (79.37)
ESR (mm/L)	15.299	<0.001		
Within normal range			50 (68.49)	22 (34.92)
Beyond normal range			23 (31.51)	41 (65.08)
IL-6 (ng/mL)	11.184	0.001		
Within normal range			52 (71.23)	27 (42.86)
Beyond normal range			21 (28.77)	36 (57.14)

### Effects of underlying diseases on the blood indices

There are more patients with underlying diseases in the severe group comparing to the mild group. Thus, further analysis according to these underlying diseases was done to examine the effects of these underlying diseases on the variation of blood indices between the two groups. By comparing between the blood index levels quantitatively and qualitatively between patients with underlying diseases in the two groups, it was found that Cr, PCT, CRP, NEUT, ESR, IL-6, PT, D-dimer were significantly higher in the severe group while levels of PLT and ALB were significantly higher in the mild group (Table 3). This showed that underlying disease is indeed a factor affecting the severity of COVID-19 infection. Blood index levels were then compared respectively according to hypertension and diabetes which are most common underlying diseases observed from the patients. Elevated levels of PCT, CRP, NEUT and decreased levels of PLT and ALB were observed from patients with diabetes in the severe group, where level of N increased both quantitatively and as a categorical variable (Table 3). For those patients with hypertension in the severe group, levels of Cr, PCT, CRP, and ESR were significantly increased while levels of PLT and ALB were decreased, where levels of CRP, ESR and ALB were varied both quantitatively and as a categorical variable (Table 3). Such results specifically highlighted the possibility of the influence of underlying diseases on the organ functions in the COVID-19 patients.

**Table 3:** Comparison of the levels of indices and the proportion with abnormality in each categorical variable between the patients with underlying diseases in the severe group and the mild group

Blood index levels in the patients with underlying diseases				
Comparison of index levels				
	Mean/Median	Mean/Median	t or Z values	P-value
Cr (μmol/L)	57.5 (51–70)	73.5 (60–102)	<0.001	0.02
PCT (ng/mL)	0.03 (0.02–0.05)	0.07 (0.05–0.12)	<0.001	<0.001
CRP (mg/L)	5 (5–13.25)	30.6 (20.2–53.98)	<0.001	<0.001
NEUT (10^9^/L)	2.3 (1.72–2.74)	2.94 (2.26–4.4)	<0.001	0.017
ESR (mm/L)	20 (15–40)	39.5 (27–52)	<0.001	0.011
IL-6 (ng/mL)	9.89±7.04	31.58±19.41	0.000	0.001
PLT (10^9^/L)	168 (152–197)	141 (121–166)	1.975	0.048
PT (s)	11.75±0.99	12.43±0.97	0	0.042
D-dimer (μg/mL)	0.4 (0.27–0.5)	0.52 (0.36–1.1)	<0.001	0.033
ALB (g/L)	43 (41–46)	39.35 (36.8–41.4)	3.451	<0.001
Categorical variable of patients with underlying diseases				
	χ^2^	P-value	Mild Group<br> No. of Patients %	Severe Group<br> No. of Patients %
Cr (μmol/L)	3.393366814	0.065459158		
Within normal range			10 (38.46)	16 (61.54)
Beyond normal range			3 (14.29)	18 (85.71)
PCT (ng/mL)	2.718145524	0.099213223		
Within normal range			13 (34.21)	25 (65.79)
Beyond normal range			0 (0)	9 (100)
CRP (mg/L)	10.88810087	0.00096784		
Within normal range			9 (64.29)	5 (35.71)
Beyond normal range			4 (12.12)	29 (87.88)
NEUT (10^9^/L)	0.43454142	0.509768915		
Within normal range			8 (23.53)	26 (76.47)
Beyond normal range			5 (38.46)	8 (61.54)
ESR (mm/L)	8.684012066	0.003210135		
Within normal range			10 (50)	10 (50)
Beyond normal range			3 (11.11)	24 (88.89)
IL-6 (ng/mL)	0.103126463	0.748109534		
Within normal range			9 (31.03)	20 (68.97)
Beyond normal range			4 (22.22)	14 (77.78)
PLT (10^9^/L)	1.01743763	0.313127583		
Within normal range			12 (32.43)	25 (67.57)
Beyond normal range			1 (10)	9 (90)
PT (s)	0.124124446	0.724603544		
Within normal range			9 (25)	27 (75)
Beyond normal range			4 (36.36)	7 (63.64)
D-dimer (μg/mL)	2.788532764	0.09494112		
Within normal range			10 (37.04)	17 (62.96)
Beyond normal range			3 (15)	17 (85)
ALB (g/L)	13.52783379	0.000235051		
Within normal range			12 (52.17)	11 (47.83)
Beyond normal range			1 (4.17)	23 (95.83)
Blood index levels in the patients with diabetes				
Comparison of index levels				
	Mean/Median<br> Mild Group	Mean/Median<br> Severe Group	t or Z values	P-value
PCT (ng/mL)	0.03 (0.02–0.03)	0.07 (0.06–0.12)	<0.001	0.011
CRP (mg/L)	12.35 (5–24.05)	31.95 (22–55.3)	<0.001	0.043
NEUT (10^9^/L)	1.65 (1.22–2.36)	2.34 (2.2–3.16)	<0.001	0.079
PLT (10^9^/L)	173.25±26.65	136.58±27.39	2.332	0.035
ALB (g/L)	43.5±4.95	38.88±4.3	1.797	0.094
Categorical variable of patients with diabetes				
		P-value (Fisher test)	Mild Group<br> No. of Patients (%)	Severe Group<br> No. of Patients (%)
PCT (ng/mL)	0.5286			
Within normal range		4 (30.77)	9 (69.23)	
Beyond normal range		0 (0)	3 (100)	
CRP (mg/L)	0.2445			
Within normal range		2 (50)	2 (50)	
Beyond normal range		2 (16.67)	10 (83.33)	
NEUT (10^9^/L)	0.0269			
Within normal range		1 (8.33)	11 (91.67)	
Beyond normal range		3 (75)	1 (25)	
PLT (10^9^/L)	0.5286			
Within normal range		4 (30.77)	9 (69.23)	
Beyond normal range		0 (0)	3 (100)	
ALB (g/L)	0.2615			
Within normal range		3 (42.86)	4 (57.14)	
Beyond normal range		1 (11.11)	8 (88.89)	
Blood index levels in the patients with hypertension				
Comparison of index levels				
	Mean/Median<br> Mild Group	Mean/Median<br> Severe Group	t or Z values	P-value
Cr (μmol/L)	63.84±9.79	80.47±29.05	0.000	0.046
PCT (ng/mL)	0.03 (0.02–0.04)	0.07 (0.04–0.17)	<0.001	0.035
CRP (mg/L)	5 (4.9–5)	29.32 (16.2–55.3)	<0.001	0.003
ESR mm/L)	20 (15–41)	40 (30–85)	<0.001	0.102
PLT (10^9^/L)	189 (176–197)	143 (122.5–168)	2.415	0.016
ALB (g/L)	44.66±2.76	38.54±4.11	3.137	0.005
Categorical variable of patients with diabetes				
	P-value (Fisher test)	Mild Group<br> No. of Patients %	Severe Group<br> No. of Patients %	
Cr (μmol/L)	0.3406			
Within normal range		4 (28.57)	10 (71.43)	
Beyond normal range		1 (9.09)	10 (90.91)	
PCT (ng/mL)	0.2743			
Within normal range		5 (27.78)	13 (72.22)	
Beyond normal range		0 (0)	7 (100)	
CRP (mg/L)	0.0123			
Within normal range		4 (57.14)	3 (42.86)	
Beyond normal range		1 (5.56)	17 (94.44)	
ESR (mm/L)	0.0377			
Within normal range		3 (60)	2 (40)	
Beyond normal range		2 (10)	18 (90)	
PLT (10^9^/L)	0.544			
Within normal range		5 (25)	15 (75)	
Beyond normal range		0 (0)	5 (100)	
ALB (g/L)	0.0149			
Within normal range		5 (41.67)	7 (58.33)	
Beyond normal range		0 (0)	13 (100)	

## Discussion

Here we analyzed the differences between the levels of organ functional indices detected from peripheral venous blood samples between two cohorts of patients with either severe or mild COVID-19 pneumonia infection. We measured the biochemical indexes and blood routine indexes in the early stage of medication in order to avoid damage the functions of patients' major organs including liver, kidney and other organs in the course of medication. Through inter-group comparison, it was found that levels of ALT, AST and Cr were significantly higher in the severe group (P<0.05), reflecting a higher vulnerability of severe patients to the damage of liver and kidney functions. Our results also indicated from the perspective of blood index levels the influence of underlying diseases on the organ functions in the severe group.

It has been reported that human coronavirus is able to replicate quickly after infection while causes extensive infiltration of inflammatory cells and enhanced responses of pro-inflammatory cytokine/chemokine [12]. Another group has also described the pathophysiological changes of COVID-19 infection using »cytokine storm«. This series of changes would lead to systemic inflammatory response syndrome and induce related organ damage in the patients. In the early stage of infection, some patients had respiratory distress syndrome and decreased lung ventilation ability which lead to hypoxia of organs and tissues. Hypoxia would result in oxidative stress responses of the body while the continuous increase of reactive oxygen species and its peroxides could further promote the release of proinflammatory factors, aggravating liver and kidney function damages [13]. Angiotensin-converting enzyme 2 (ACE-2) receptors are distributed over the cells surface of brain, heart, kidney, liver, lungs and etc. Being a linear single positive strand RNA virus, COVID-19 virus infects human cells by interacting with ACE-2 receptors through RBS domains of S proteins inducing a series of pathological changes and related organ function damages [14]. Our results also confirmed that patients in the severe group had significantly altered level of inflammatory indices including lymphocyte count, PCT, CRP, ESR, IL-6 and etc., and higher proportion of bilateral lung involvement, comparing to those in the mild group. This also indicates that severe patients are more prone to have inflammatory storm and hypoxia of respiratory distress tissue.

Moreover, patients in the severe group have higher average age and more chronic diseases which make them more vulnerable to liver and kidney function damages under the influence of inflammatory storm comparing to the patients in the mild group. There are several studies reported on the organ damage in the COVID-19 patients. Cui et al. [15] has reported a case of female infant with COVID-19 pneumonia presented damages on liver and heart, diagnosed by laboratory examinations. Liver dysfunctions as indicated mostly by increased AST levels in the COVID-19 patients were also reported. The mechanisms of the induction of these liver dysfunctions were postulated due to mediation of ACE2, inflammatory responses, hypoxia and even medications [16]. Our group has also reported one severe patient with onset of viral hepatitis after confirmed with COVID-19 infection [17]. Kidney damages related to COVID-19 were possibly induced in the patients by cytokine storm, organ crosstalk and Systemic effects [18]. This disease is also more severe in the immunocompromised kidney transplant recipient [19]. COVID-19-related myocardiac injury was due to SARS-CoV-2 invasion mediated by ACE-2 [20]. One case in the severe group had definite myocardial injury which is resulted from viral myocarditis caused by COVID-19 virus. There is no difference statistically in the levels of CK-MB and TnI in other severe patients comparing to those in the mild group (P>0.05). Further dynamic observation and analysis are still needed due to the factors of sample size and collection time of peripheral blood. In summary, patients with severe COVID-19 pneumonia infection are possibly associated with different organ damages in the early stage, such as in the lung, the liver, the kidney and etc. Therefore, it is necessary to dynamically monitor the relevant organ function indices for prompt drug intervention, prevention of aggravation of patients' conditions and improvement of prognosis.

## Dodatak

### Funding

This work is supported by Natural Science Foundation of China (81672003) and National Science and Technology Major Project (2017ZX10103011), and Shenzhen High-level Hospital Construction Fund (No.SZGSP011).

### Conflict of interest statement

All the authors declare that they have no conflict of interest in this work.
